# Determination of Odour Interactions in Gaseous Mixtures Using Electronic Nose Methods with Artificial Neural Networks

**DOI:** 10.3390/s18020519

**Published:** 2018-02-08

**Authors:** Bartosz Szulczyński, Krzysztof Armiński, Jacek Namieśnik, Jacek Gębicki

**Affiliations:** 1 Department of Chemical and Process Engineering, Faculty of Chemistry, Gdańsk University of Technology, 11/12 G. Narutowicza Str., 80-233 Gdańsk, Poland; 2 Department of Control Engineering, Faculty of Electrical and Control Engineering, Gdańsk University of Technology, 11/12 G. Narutowicza Str., 80-233 Gdańsk, Poland; krzysztof.arminski@gmail.com; 3 Department of Analytical Chemistry, Faculty of Chemistry, Gdańsk University of Technology, 11/12 G. Narutowicza Str., 80-233 Gdańsk, Poland; jacek.namiesnik@pg.edu.pl

**Keywords:** electronic nose, odour interactions, odour intensity, hedonic tone, artificial neural networks

## Abstract

This paper presents application of an electronic nose prototype comprised of eight sensors, five TGS-type sensors, two electrochemical sensors and one PID-type sensor, to identify odour interaction phenomenon in two-, three-, four- and five-component odorous mixtures. Typical chemical compounds, such as toluene, acetone, triethylamine, α-pinene and n-butanol, present near municipal landfills and sewage treatment plants were subjected to investigation. Evaluation of predicted odour intensity and hedonic tone was performed with selected artificial neural network structures with the activation functions tanh and Leaky rectified linear units (Leaky ReLUs) with the parameter a=0.03. Correctness of identification of odour interactions in the odorous mixtures was determined based on the results obtained with the electronic nose instrument and non-linear data analysis. This value (average) was at the level of 88% in the case of odour intensity, whereas the average was at the level of 74% in the case of hedonic tone. In both cases, correctness of identification depended on the number of components present in the odorous mixture.

## 1. Introduction

Odour intensity and hedonic tone of odorants mixture above a threshold level are often different from the quantities predicted based on concentration of particular components and their odour thresholds. Odorant mixtures are characterized by odour interactions. They include synergism, masking and neutralization [1,2,3,4,5]. Synergism is mutual amplification of two or more stimuli. Masking is usually a substitution of an unpleasant odour with another, more pleasant one. Neutralization, also called compensation, is typically interpreted as activity of additional component of the odorants mixture, which leads to disappearance of odour or to distinct decrease in its intensity. Investigations on odour interactions have been carried out for many years, however, they have not led to explanation of the mechanisms of these processes so far. Odour interaction models (perception models) are analysed in the case of odour interactions presence. They describe dependence between the odour intensity of air containing pollutants mixture and the odour intensity, which would be exemplified if these pollutants were present separately. A multitude of these models engulfs Zwaardemaker, Berglund, Patte and Laffort or U models [6,7,8,9]. However, the investigated objects are usually mixtures containing two or three types of odorants. In actual conditions, odorant mixtures are much more complex.

Odour analysis techniques, especially the ones that utilize holistic analysis, which skips the stage of mixture separation into its particular components, include olfactometric and electronic nose (e-nose) methods. Human nose is a sensor describing odour intensity or hedonic tone in the case of olfactometric technique. As far as e-nose is concerned, the chemical sensors, belonging to different classes and characterized by various metrological parameters, are the counterparts of olfactory receptors [10,11,12,13,14]. Additionally, the e-nose technique requires a training stage to provide proper operation of a device [15,16,17]. The advantages of the e-nose instruments, as compared to the olfactometric techniques, include: possibility of on-line measurements, possibility to record the episodes of severe and short-duration odour nuisance, no need for olfactory adaptation and no need for qualified personnel, who are indispensable in the case of olfactory measurements.

Different techniques of training under supervision are utilized for this purpose. They are employed for construction of different types of models, such as regression, discrimination or classification models, depending on the research problem under investigation. The most popular regression models include multiple linear regression [18,19], principal component regression [20,21], partial least squares [22,23] or artificial neural networks (ANNs) in the case of non-linear models. To improve the regression model, one has to use a set of explaining variables and a set of dependent variables. The set of explaining variables contains the information from the chemical sensors comprising an e-nose device. The set of dependent variables includes the values of odour intensity or hedonic tone expressed in the verbal scale, which originate from a group of assessors utilizing suitable olfactometric technique. A task of the regression methods is to construct such a model, which would allow quantitative evaluation of particular odour feature (odour intensity, hedonic tone) based on the set of explaining variables.

ANNs are applied as a tool for interpretation of the data, obtained from the electronic nose-type devices [24,25,26,27,28,29]. ANNs were used for prediction of odour intensity of two-component mixtures [30], whereas principal component regression model was employed to estimate odour intensity and hedonic tone of three-component mixtures [31]. Prediction of odour intensity, hedonic tone or odour interactions in multi-component mixtures is a difficult task and it calls for application of non-linear methods. These requirements can be fulfilled by ANNs with sufficient number of neurons in a given layer or suitable activation function. This paper describes attempts to determine odour interactions of gas two-component, three-component, four-component and five-component mixtures, exhibiting different character of odour, employing a prototype of e-nose comprised of eight sensors of three types: five semiconductor sensors, two electrochemical sensors and one photoionization detector (PID). Investigated chemical compounds, being the components of odour mixture, are typical odorous compounds present in gas odour mixtures in a vicinity of municipal landfills or sewage treatment plants. Additional aim of the investigation is the attempt of odour measurement instrumentation as well as acquisition of the information about e-nose instruments as devices for air quality monitoring with respect to odour nuisance. The paper adheres to the trend, represented by the working group CEN/TC264/WG41, searching for a new European standard of instrumental monitoring of malodours [32].

Selected ANN structures were used to determine presence of synergism phenomenon of odour intensity and hedonic tone with respect to the theoretical values estimated with the Patte and Laffort model. Proposed regression models were verified with coefficient of determination (R^2^) and root mean square error of prediction (RMSEP).

## 2. Materials and Methods

### 2.1. Types of Gas Odorous Mixtures and Their Preparation

The investigation employed the following pure substances and their mixtures: toluene, acetone, triethylamine, α-pinene and n-butanol. Table 1 presents characteristics of the investigated odorous compounds, including odour type, vapour pressure, olfactory threshold in gas phase and olfactory threshold in aqueous solution [33,34].

**Figure 1:**
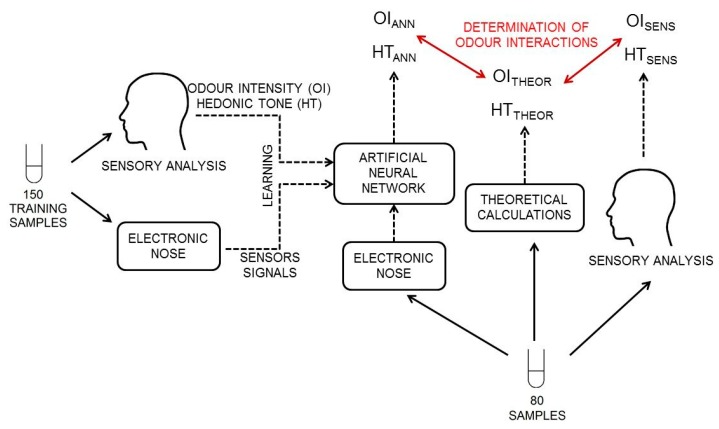
Scheme of the process of determination of synergism phenomenon for odour intensity and hedonic tone using artificial neural network.

The first step involved preparation of 150 samples of aqueous solutions of two-, three-, four- and five-component mixtures, characterized by odour intensity from 0.5 to 5.5 and by hedonic tone from −3.5 to 1. The values of odour intensity and hedonic tone were determined using the scale proposed in the document VDI 3940. Concentrations of particular compounds in these aqueous solutions were as follows: toluene (5–80 ppm *v*/*v*), acetone (200–3200 ppm *v*/*v*), triethylamine (5–80 ppm *v*/*v*), α-pinene (1–16 ppm *v*/*v*) and n-butanol (20–320 ppm *v*/*v*). The samples were subjected to sensory analysis by a panel of assessors. They also underwent investigation with the prototype of e-nose. The information obtained from both investigations was utilized during training of the ANN for different architecture scheme and activation function to select the structure of the ANN, characterized by the lowest values of root mean square error of cross-validation (RMSECV) and determination coefficient (R^2^). The next step involved preparation of new 80 samples of aqueous solutions of two-, three-, four- and five-component mixtures, characterized by odour intensity from 0.5 to 5.5 and by hedonic tone from −3.5 to 1. These samples exhibited the same concentration ranges of particular components of the mixtures as the ones used for training of ANN. This stage of research was aimed at utilization of already selected ANN to predict the values of odour intensity and hedonic tone for particular samples. Another aim was evaluation, based on a comparison with the theoretical values, which samples exhibited synergism. The entire process of synergism detection using ANN is schematically presented in Figure 1.

### 2.2. Measurements of Odour Intensity and Hedonic Tone

The investigation was carried out by a group of assessors whose task was olfactory evaluation of prepared samples: 150 specimens for test examinations and 80 specimens for examinations of odour interactions. This group consisted of four persons, trained according to a procedure elaborated by St. Croix Sensory, Inc. (St. Croix Sensory 2006, Stillwater, MN, USA). 

The task of each assessor was determination of odour intensity and hedonic tone of prepared samples of aqueous solutions. Each sample was attributed the odour intensity within the range from 0 to 6 and the hedonic tone from −4 to 4, following the scale of odour intensity and hedonic tone proposed in the document VDI 3940.

### 2.3. Description of Experimental Setup for Electronic Nose Investigations

**Figure 2:**
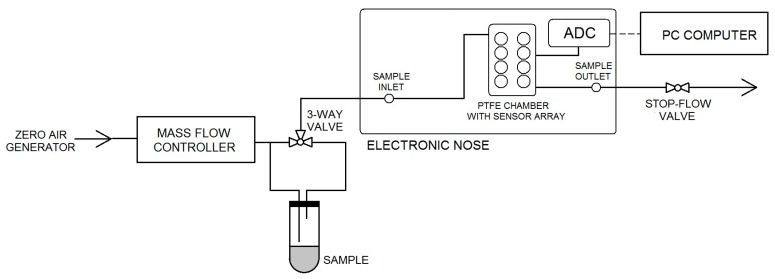
Scheme of experimental setup containing electronic nose prototype.

A scheme of the experimental setup is demonstrated in Figure 2. It consisted of:

- zero air generator
- mass flow controller
- three-way valve
- sample mounting system
- prototype of e-nose equipped with a matrix of eight sensors: five MOS-type sensors, two electrochemical sensors by Figaro Co. and one PID-type sensor (Table 2)
- analogue-to-digital converter
- PC-class computer

### 2.4. Methodology of Measurement Using Electronic Nose

Air from the generator was flowing through a measurement system. A three-way valve was connected with the chamber containing investigated sample to enable air flow through the sample as well as in a by-pass mode. The first tube provided air to the sample, whereas the second tube led aerated phase into a measurement sensors chamber of the e-nose. Recording of signal started 50 s after the moment, when air had been passed through the probes with investigated substances. The signal was recorded for 30 s. After that time, the three-way valve was turned into the by-pass mode, which enabled cleaning of the measurement system. Cleaning occurred due to undisturbed flow of the carrier gas until e-nose signal returned to the initial level. The measurement parameters were determined via optimization method and they were as follows:

- volumetric flow rate of air, determined using the mass flow controller: 0.3 L/min;
- time of carrier gas flow through the sample: 50 s; and
- signal recording: 30 s.

The system operated in a stop–flow mode, meaning 50 s of carrier gas flow, then 30 s of carrier gas flow interruption, and finally 8 min of carrier gas flow. The mixture of odorous compounds with air was supplied to the measurement chamber of the e-nose at the temperature 20 °C ± 1 °C and the relative humidity 60 ± 5%. The measurement was conducted with temperature and humidity sensor exposed to the flow path before the sensors chamber. A total of 230 samples were investigated to determine odour intensity and hedonic tone.

### 2.5. Data Analysis and Artificial Neural Network Structure

Selected measurement values from the sensors constitute the inputs to the network ($u\in \Omega_{in}={\mathbb{R}}^{8}$), whereas the values obtained with sensory analysis, intensity (OI) and hedonic tone (HT) are the outputs. The research presented in the paper involved designing of two independent networks differing in an adopted output. In case of the first one (ANN_OI_), the output value will be intensity ($y_{OI}\in {\mathbb{R}}^{1}$), the output of the second network (ANN_HT_) will be hedonic tone ($y_{HT}\in {\mathbb{R}}^{1}$).

**Figure 3:**
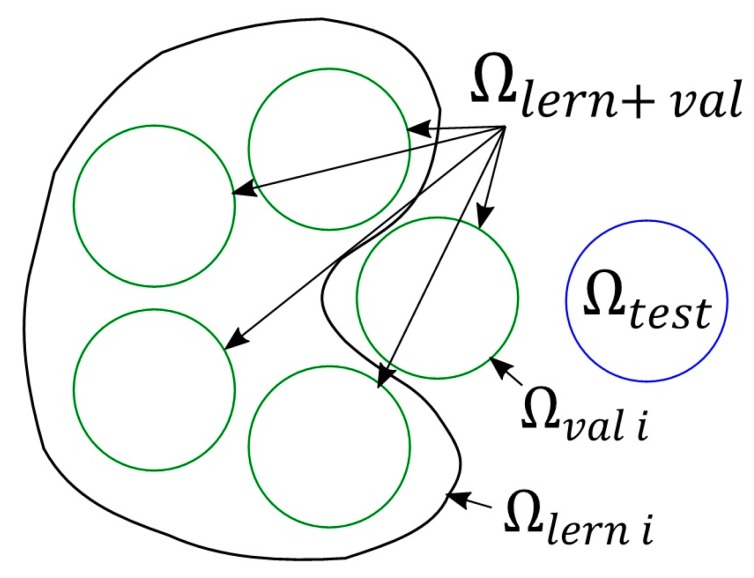
Data division.

As mentioned before, both networks will solve regression task, which requires the output scale to be extended from single classes to continuous values. Even though during evaluation odour parameters are assigned to single classes by particular assessors, it was decided to utilize their average values to generate the output. Since the results obtained from ANN need verification, a set of learning data and a set of testing data were separated from the input data, where $\Omega_{lern}\cap^{\text{​}}\Omega_{test}=\emptyset$. Unfortunately, utilization of the testing set to determine network structure can cause that the network will adjust to a specificity of the testing set, instead of general features of the problem upon a process of structure selection. Hence, it is necessary to employ additional data set (validation one) to describe desired ANN structure. The expense of this approach is a decrease in the number of samples used for network training. To prevent this, it was decided to utilize K-Folds cross validation technique [35]. In this method, the first step consists in separation of the testing set ($\Omega_{test}$) only, whereas the remaining samples ($\Omega_{lern+val}$) are divided into *n* parts. Then, the network with given parameters is trained *n* times, each time utilizing n−1 parts as the learning sets ($\Omega_{lern\;i}$, $i\in \bar{1,n}$) and the last part as the validation set ($\Omega_{val\;i}$). This division is presented in Figure 3.

At the validation stage, each network will provide different ANN evaluation, thus they are converted into a single value via averaging. Quality evaluation of selected structure is obtained in this way. Following selection of suitable structure, direct data from $\Omega_{lern+val}$ are used for network training. An increase in amount of data usually leads to a decrease in demand for network regularization, so the selected structure should not require additional regularization, providing comparable results.

As each network possesses only one output, its normalization cannot be omitted, which allows easier interpretation of particular network quality indicators, especially comparing it to the value of 1, being a difference between particular classes. 

As far as the network inputs are concerned, in both cases, to improve convergence of an optimization algorithm, it was decided to perform normalization to accelerate the ANN training process [36,37]. This action was defined in affine way, equivalently it can be performed in the first layer of the network without a need for increased number of necessary calculations via change of the weights values. This task was accomplished through deletion of average value according to:

(1) $$u_{n}≜u_{n}\left(u\right)≜u-\underset{u_{k}\in \Omega_{lern+val}}{\sum }u_{k}$$

where $u_{n}\in \Omega_{\mathrm{in}}$ is the input after the first normalization stage and i is the number of sample and normalized variance:

(2) $$u_{nn}≜u_{nn}\left(u_{n}\right)≜u_{n}\frac{\overset{=}{\Omega_{n\;lern+val}}}{\sum_{u_{k}\in \Omega_{n\;lern+val}}{\left(u_{n\;i}\right)}^{2}}$$

where $u_{nn}$ is the normalized network input.

Accordingly, demanded static models in the form $f_{model}:\Omega_{in}\to \mathbb{R}$ will be the effect of the combination $f_{model}\left(u\right)=f_{ANN}\left(u_{nn}\left(u_{n}\left(u\right)\right)\right)$ where $f_{ANN}:{\mathbb{R}}^{8}\to \mathbb{R}$ is the function described using designed ANN.

Then model quality indicator was accepted as:

(3) $$MSE≜\frac{1}{n}\overset{n}{\underset{i=1}{\sum }}{\left(\hat{y_{i}}-y_{i}\right)}^{2}$$

where $y_{i}$ and $\hat{y_{i}}$ are the actual value of the output and the model output corresponding to the input $u_{nn\;i}$, respectively, n is the amount of output data, and

(4) $$R^{2}≜1-\frac{\sum_{i=1}^{n}{\left(\hat{y_{i}}-y_{i}\right)}^{2}}{\sum_{i=1}^{n}{\left(y_{i}-\frac{1}{n}\sum_{i=1}^{n}y_{k}\right)}^{2}}$$

#### 2.5.1. Architecture

The problem under consideration requires selection of adequate architecture of ANN. The problem of regression is of static nature with non-linear structure, for which feedforward ANN is a suitable tool. The solutions of such type allow mapping of the relation with accuracy dependent on adopted structure [38].

ANN is built of a number of layers, each defined by the non-linearity introducing activation functions $f_{ac\;i}:{\mathbb{R}}^{n_{i}}\to {\mathbb{R}}^{n_{i}}$, and a matrix of thresholds and weights $w_{i}\in {\mathbb{R}}^{n_{i}\times \left(n_{i-1}+1\right)}$, where $n_{i}$ is the number of neurons in the *i*-th layer. Output value of each layer is defined as:

(5) $$y_{i}=f_{ac\;i}\left(z_{i}\right),\;\mathrm{where}\;z_{i}=w_{i}\left[\begin{array}{c} 1\\ y_{i-1} \end{array}\right]$$

where $y_{0}=u_{nn}$ is the input layer. Accordingly, designing of the structure requires selection of the number of layers, number of neurons present in the layers and activation functions, whereas the parameters are set with respect to suitable procedure of the network training. Then, each of the architectures should be validated, which allows selection of the best option. In the case of all networks under consideration, the activation function was assumed linear function for the initial layer, whereas differentiable functions of “tanh” type [36] and differentiable, but not in all points, “Leaky ReLUs” function [39] were considered for hidden layers (between the initial and final ones). Number of neurons and number of layers influence the level of complexity of the function under consideration. Experimental investigation was aimed at identifying which system is more advantageous.

#### 2.5.2. Training

**Figure 4:**
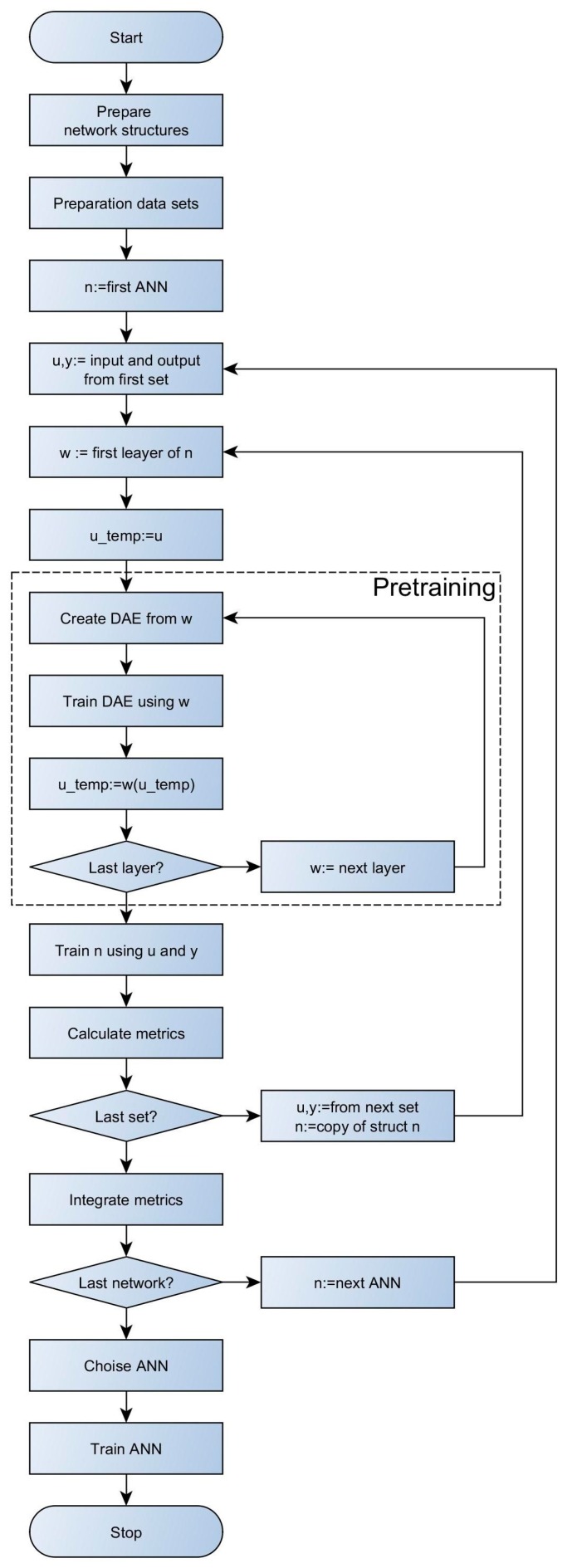
Procedure of network selection and its tuning.

Finding proper weight factors of ANN is a challenging task [36,40]. Due to non-linearity, one can encounter several local minima during tuning, which renders it difficult to find the global minimum using the gradient methods. That is why, some actions can be undertaken to improve the quality of the minimum identification [41]. One of them assumes that the local minima can also be used as a regularization mechanism. This approach utilizes that fact that initialization of the weights determines which of the minima will be obtained during the training stage [42]. In this paper, the weights will be pre-tuned, prior to the actual training, using Stacked Denosing Autoencoder (SDAE) [43,44]. This procedure is presented in Figure 4.

Pre-training is based on non-supervised teaching and is effective, as it allows to transmit important information to deeper layers, making the actual training more efficient. The pre-training was performed for each layer separately via construction of additional network for tuning of Denosing Autoencoder (DAE) type. An input to DAE is the data obtained from the previous layer, acquired after its pre-tuning (or the input data to the system in case of the first layer). Pre-training in DAE consists in nosing of data and their transmission to the investigated layer and additionally to the layer, which contains the number of neurons equal to the number of DAE inputs. Then, the obtained outputs are compared with DAE inputs and resulting error is minimized in a tuning procedure via selection of the weights using adequate algorithm. The tuning, in the case of both pre-training and actual training of the network, was carried out using an algorithm with an adaptive step to limit the number of controlling parameters.

Table 3 presents the ANN structures, which were selected for training on 150 samples of two-, three-, four- and five-component aqueous mixtures, characterized by odour intensity from 0.5 to 5.5 and hedonic tone from −3.5 to 1. Additionally, tanh and Leaky ReLUs were used as the activation function.

## 3. Results and Discussion

### 3.1. Selection of Optimum Artificial Neural Network Structures

Table 4 and Table 5 present the results, in the form of RMSECV and R^2^, obtained during training of the ANN structures mentioned in Table 3. The ANN with structure 8-3-3-1 and activation function tanh is characterized by the best parameters for determination of odour intensity of investigated odorous mixtures. Concerning hedonic tone of the odorous mixtures, the superior parameters are exhibited by the ANN with structure 8-3-2-1 and activation function Leaky ReLU with the parameter a=0.03 (the parameter a was determined via optimization during ANN training process).

Only the ANN with the best parameters were employed in further investigations, aimed at determining the occurrence of odour interactions (synergism phenomenon) in examined odorous mixtures.

### 3.2. Validation of Selected Artificial Neural Network Structures on Test Samples

**Figure 5:**
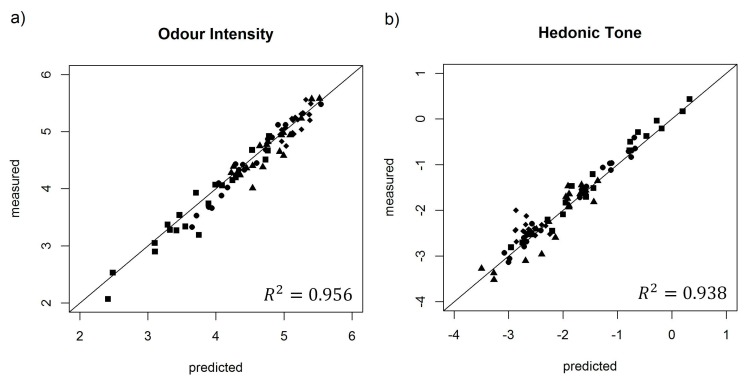
(**a**) Model of artificial neural network with structure 8-3-3-1 and activation function tanh presented as predicted odour intensity with respect to the odour intensity determined experimentally by the group of assessors. (**b**) Model of artificial neural network with structure 8-3-2-1 and activation function Leaky ReLUs with the parameter a=0.03 presented as predicted hedonic tone with respect to the hedonic tone determined experimentally by the group of assessors. ∎, two-component; ●, three-component; ▲, four-component; ◆, five-component.

The ANN structures, which were selected for determination of odour intensity and hedonic tone in the previous stage, were then verified with respect to detection abilities of odour intensity and hedonic tone of new 80 samples of two-, three-, four- and five-component aqueous mixtures. These specimens were characterized by different values of odour intensity and hedonic tone from the 150 samples utilized for the ANN training. Figure 5a presents dependence between predicted odour intensity for two-, three-, four- and five-component mixtures, obtained based on the ANN with structure 8-3-3-1 with activation function tanh, and the odour intensity observed experimentally for these mixtures. The figure illustrates that proposed ANN structure with given activation function provides very good prediction of the value of odour intensity for two-, three-, four- and five-component samples as compared to the values of odour intensity determined by the group of assessors. Coefficient of determination of this dependence R^2^ is high and equal 0.956. The ANN structure, proposed for prediction of hedonic tone of the aforementioned odorous mixtures, was characterized by high value of correlation at the level of $R^{2}=0.938$ (Figure 5b). It is evidence that the first stage yielded suitable ANN structures with appropriate activation functions for assessment of odour intensity and hedonic tone.

Table 6 presents root mean square error of prediction (RMSEP) for determination of odour intensity and hedonic tone for two-, three-, four- and five-component mixtures, using the ANN structures selected during the ANN training stage. An increase in the number of components of odorous mixture is accompanied by an increase in RMSEP value for both odour intensity and hedonic tone. Odour intensity parameter was characterized by lower RMSEP values. The most probable reason is the fact that sensor signal directly depends on concentration of particular component and the concentration of a given component is also directly connected with odour intensity. In the case of hedonic tone, this dependence is not directly connected with the signal of given sensor, which generates inferior prediction values.

### 3.3. Determination of Odour Interactions Using Selected Artificial Neural Network Structures

To determine theoretical value of odour intensity of 80 samples of two-, three-, four- and five-component mixtures, the Patte and Laffort model was used (which adopts Euclidean additivity of particular components of the mixture when present separately [8]):

(6) $$OI_{mix}=\sqrt{OI_{1}^{2}+OI_{2}^{2}+OI_{3}^{2}+OI_{4}^{2}+OI_{5}^{2}}$$

To determine theoretical value of hedonic tone of 80 samples of two-, three-, four- and five-component mixtures, the algebraic summation was used:

(7) $$HT_{mix}=\overset{5}{\underset{i=1}{\sum }}HT_{i}$$

Independently of determined theoretical values of odour intensity and hedonic tone, the group of assessors conducted the investigations aimed at evaluation of odour intensity and hedonic tone of 80 samples of two-, three-, four- and five-component mixtures. Moreover, four samples were examined with the e-nose prototype. In this way, it was possible to identify these multi-component mixtures, where odour interaction phenomenon occurred.

**Figure 6:**
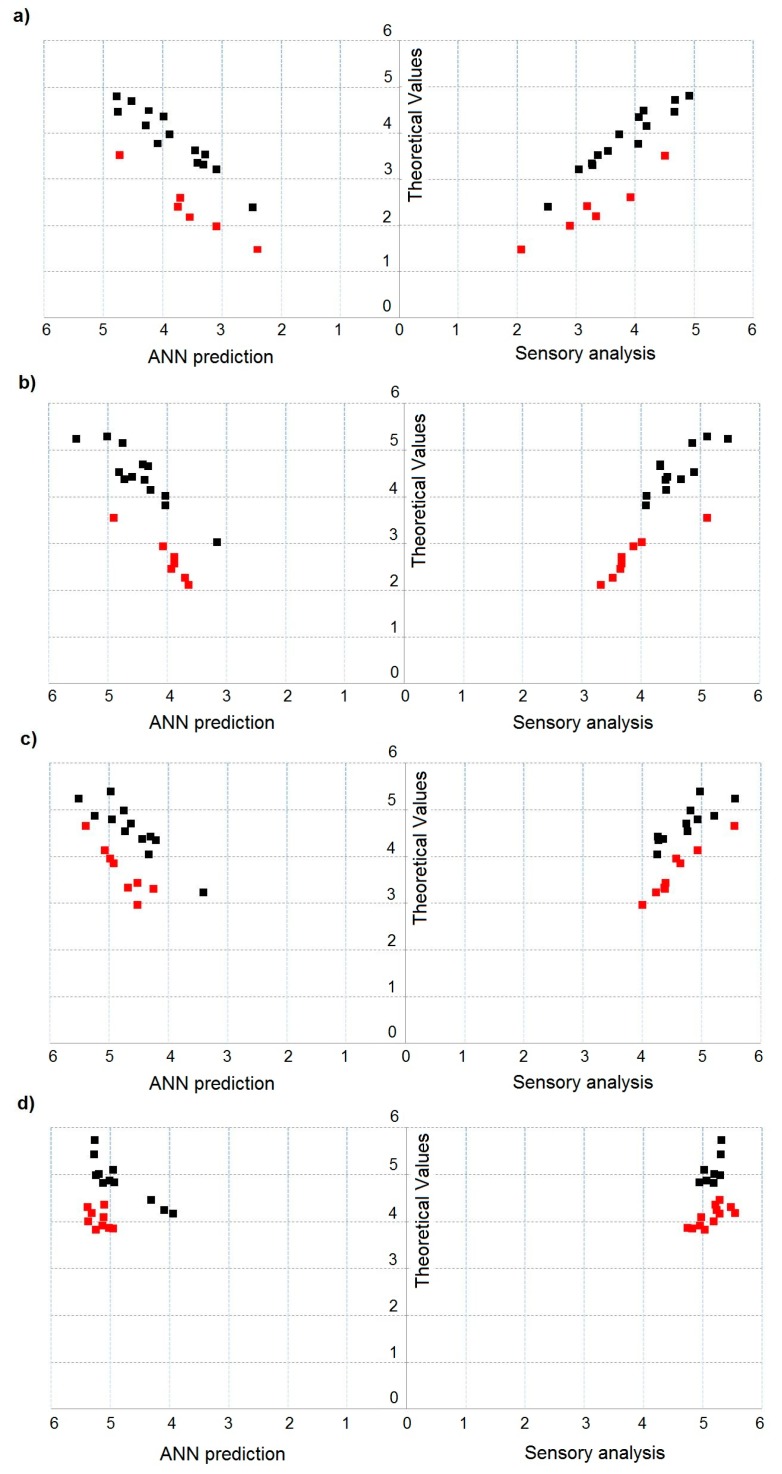
Dependence between theoretical value of odour intensity and predicted odour intensity determined using artificial neural network with structure 8-3-3-1 and activation function tanh and experimental odour intensity evaluated by the group of assessors: (**a**) for two-component mixture; (**b**) for three-component mixture; (**c**) for four-component mixture; and (**d**) for five-component mixture.

Figure 6a–d depicts dependence between theoretical value of odour intensity and predicted value of odour intensity, determined using the ANN with structure 8-3-3-1 and activation function tanh, and experimental odour intensity evaluated by the group of assessors for two- (Figure 6a), three- (Figure 6b), four- (Figure 6c) and five-component (Figure 6d) mixtures. Red points indicate multi-component mixture samples, for which theoretical values differed statistically significantly from the values predicted with the ANNs and the experimental values. An increase in the number of components of odorous mixture is associated with an increase in the number of samples differing in odour intensity at statistically significant level. 

**Figure 7:**
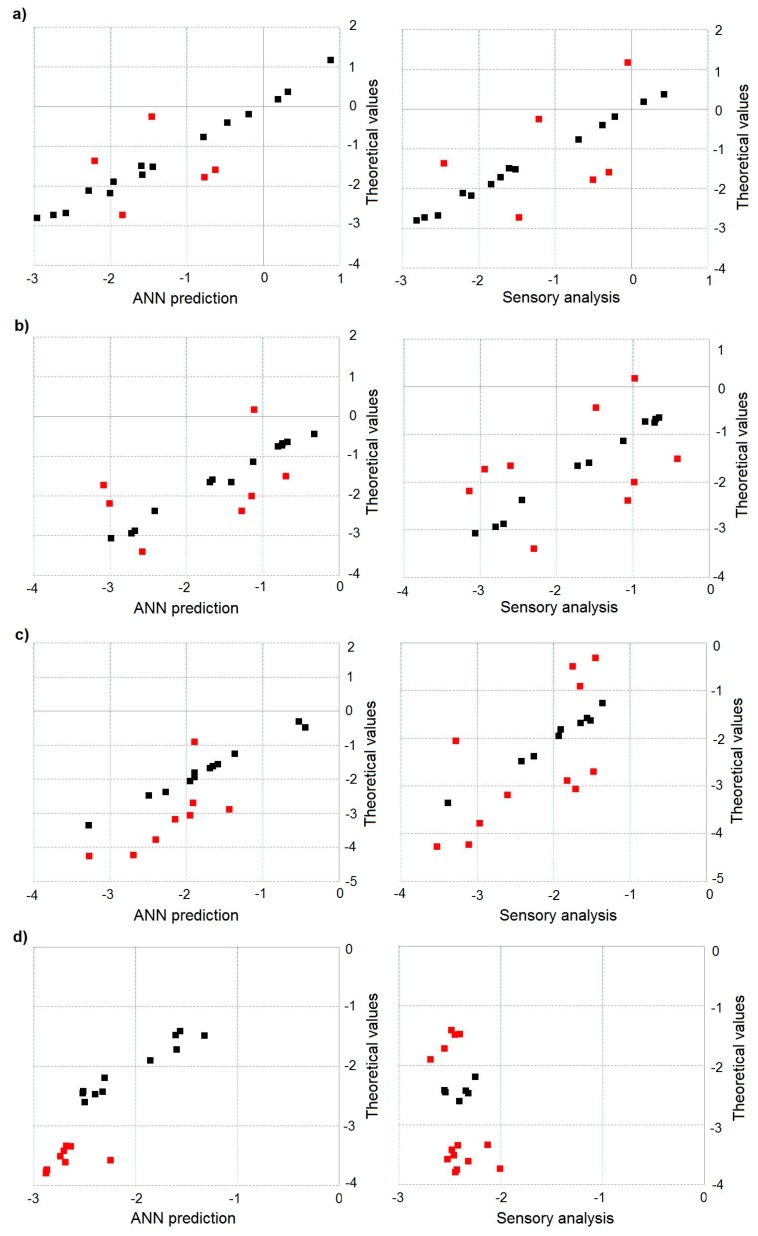
Dependence between theoretical value of hedonic tone and predicted hedonic tone determined using artificial neural network with structure 8-3-2-1 and activation function Leaky ReLUs with the parameter a=0.03 and experimental hedonic tone evaluated by the group of assessors: (**a**) for two-component mixture; (**b**) for three-component mixture; (**c**) for four-component mixture; and (**d**) for five-component mixture.

Moreover, in all cases (multi-component mixtures), there is synergism phenomenon, meaning odour intensity amplification. Figure 7a–d illustrates dependence between theoretical value of hedonic tone and predicted value of hedonic tone, determined using the ANN with structure 8-3-2-1 and activation function Leaky ReLUs with the parameter a=0.03, and experimental hedonic tone evaluated by the group of assessors for two- (Figure 7a), three- (Figure 7b), four- (Figure 7c) and five-component (Figure 7d) mixtures. Again, red points indicate the multi-component mixture samples, for which theoretical values differed statistically significantly from the values predicted with the ANN and the experimental values. Similar to evaluation of odour intensity, it can be noticed that an increase in the number of components of odorous mixture is accompanied by an increase in the number of samples differing in hedonic tone at statistically significant level. However, unlike in the case of odour intensity assessment, in these samples one could observe not only synergism phenomenon but also neutralisation, meaning attenuation of hedonic odour. It was caused by presence of α-pinene in the multi-component mixtures, which is characterized by pleasant odour that neutralized the odours originating from the remaining chemical compounds, present in the odorous mixtures.

Table 7 shows the number of odorous samples, where both types of investigation (sensory analysis and e-nose prototype) revealed presence of odour interactions. The table also contains the information about percentage of correct identifications obtained with both methods. Correctness of odour interactions identification for odour intensity was 100% in case of two-component mixtures, 88% in three-component mixtures, 89% in four-component mixtures and 75% in five-component mixtures. In the case of odour interactions for hedonic tone, the respective percentage was as follows: 83% for two-component mixtures, 78% for three-component mixtures, 73% for four-component mixtures and 64% for five-component mixtures. Obtained results confirm the rule that evaluation of odour intensity is more accurate than evaluation of hedonic tone, when speaking about e-nose measurements.

Literature provides papers about investigations on odour activity value (OAV), which is widely applied to evaluation of air pollution with malodorous compounds. Wu et al. [45] comments on variability of OAV and potential, inaccurate evaluation of contribution of particular odorants to odour interaction effect between mixture components. Interaction in two-component mixtures was evaluated. It was revealed that utilization of odour activity factor (OAF) was advantageous to determine actual OAV. The correlation between OAF sum and odorants concentration was at the level of 80%. Wu et al. [46] shows that sum of odour intensities (SOI) and equivalent odour concentration (EOC) can be used for air quality evaluation with respect to odour. These parameters utilize not only olfactory thresholds, but also k coefficient from the Weber-Fechner equation to provide correct odour evaluation. Yan et al. [47] proposes a model of odour interactions for two-component mixtures of benzene and its derivatives employing partial differential equation (PDE). Application of this method enabled combination of odour intensity of a mixture with individual odour activity value of an odorant. Obtained results showed that the PDE constituted an easy for interpretation method, which related particular components to summary odour intensity. The aforementioned examples indicate that the investigations on mutual relations and interactions between components in odorous mixtures are still being conducted. Determination of odour interactions between the components of multicomponent mixtures using instrumental devices is still difficult and complex task.

### 3.4. Influence of Particular Sensors on the Information Acquired from Investigated Odorous Mixtures

**Figure 8:**
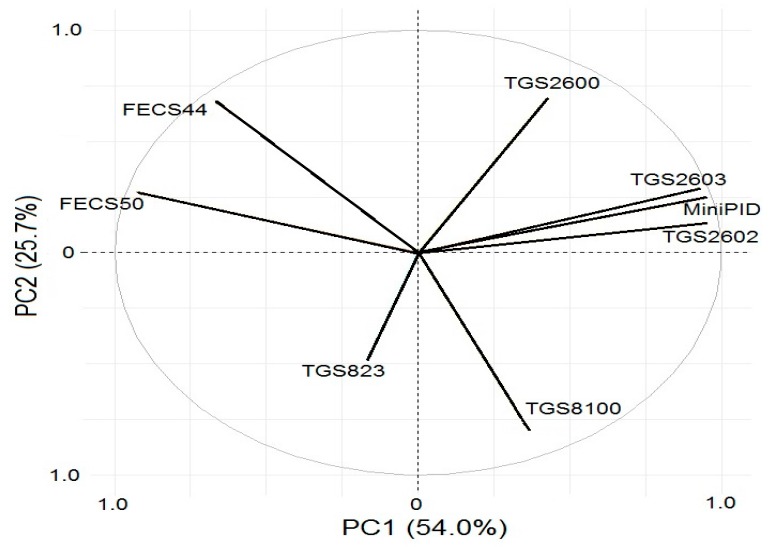
Influence of particular sensors on the information acquired from investigated odorous mixtures.

To investigate, which of the parameters are similar and which discriminate the investigated sample, a projection of the weights on the planes defined by pairs of principal components is done using PCA method. Mutual similarities are described based the angle between two vectors of the weights with the origin in [0, 0] point and the terminals defined by corresponding values of the weights of variables. If the angle between two parameters is 0, they reveal strong positive correlation. If the angle between two vectors is close to 180, the parameters show strong negative correlation. Two parameters are independent (orthogonal) if the angle between them is close to 90. Figure 8 shows that significant contribution to the first component originates from the sensors TGS2602, MiniPID, TGS2603, FECS44, and FECS50, because absolute values of their weights (a projection of vector on the first component axis) are the biggest. The conclusion is that application of these five sensors is sufficient for investigation of odour interactions in the mixtures with 2–5 components.

## 4. Conclusions

The authors of the paper propose employing an e-nose prototype as an instrumental tool, consisting of semiconductor sensors, electrochemical sensors and a PID-type sensor, to verify whether it is possible to observe odour interaction phenomenon in two-, three-, four- and five-component odorous mixtures. Selected ANN structure with suitable activation function is proposed as a data analysis tool. The ANN with structure 8-3-3-1 and activation function tanh is used for evaluation of odour intensity of samples. The ANN with structure 8-3-2-1 and activation function Leaky ReLUs with the parameter a=0.03 is utilized for evaluation of hedonic tone of the samples. The results provided by the group of assessors as well as the e-nose measurements reveal that odour interactions occur in selected multi-component mixtures. The more components there are in the mixture, the stronger are the interactions that take place. Application of the ANN with suitable structure and activation function is more successful in determining the odour interactions when odour intensity is evaluated than when hedonic tone is predicted. Average correctness of odour interactions determination, using the e-nose and appropriate ANN structure, is at the level of 88% for odour intensity and 74% for hedonic tone, obviously depending on the number of components in the odorous mixture. The mixtures of odorous compounds in this investigations include typical chemical compounds present in municipal landfills or sewage treatment plants. Odorous mixtures are selected randomly to show that it is possible to observe odour interactions with instrumental tools, such as the e-nose. This technique can supplement olfactometric techniques as well as be used for ambient air quality evaluation with respect to presence of odour nuisance.

## Figures and Tables

**Table 1:** Characteristics of investigated odorous substances.

Substance	Odour Type	Vapour Pressure (hPa)	Olfactory Threshold in Gas Phase (ppm *v*/*v*)	Olfactory Threshold in Aqueous Solutions (ppm *w*/*v*)
toluene	pleasant and characteristic	29	0.33–2.9	0.042
acetone	fruity, sweet	233	13–100	20
triethylamine	fish, pungent	72	0.000032–0.48	0.42
n-butanol	rancid, sweet	8	0.038–1	7.1
α-pinene	pine, resinous	5	0.018	4.2

**Table 2:** Types of chemical sensors and their metrological parameters used to build an electronic nose prototype.

Sensor Type	Model/Manufacturer	Target Gases	Typical Detection Range	Sensitivity
PID	MiniPID/ION Science	VOCs	1 ppb–40 ppm isobutylene	25 mV/ppm
EC	FECS44-100/Figaro	Ammonia	1~100 ppm	0.1 µA/ppm
EC	FECS50-100/Figaro	Hydrogen Sulphide	1~100 ppm	0.7 µA/ppm
MOS	TGS2600/Figaro	Air contaminants	1~10 ppm H_2_	0.3~0.6 ^a^
MOS	TGS823/Figaro	Organic Solvent Vapours	50~1000 ppm n-hexane	0.4 ± 0.1 ^a^
MOS	TGS2602/Figaro	Gaseous air contaminants (VOCs and odorous gases)	1~30 ppm EtOH	0.15~0.5 ^a^
MOS	TGS2603/Figaro	Air contaminants (trimethylamine, methylmercaptan, etc.)	1~30 ppm EtOH	~0.5 ^a^
MOS	TGS8100/Figaro	Air contaminants (hydrogen, ethanol, etc.)	1~30 ppm H_2_	~0.6 ^a^

^a^ Sensors resistance in detected gas divided by sensors resistance in the air.

**Table 3:** Artificial neural network structures used for training on 150 samples of two-, three-, four- and five-component aqueous mixtures characterized by odour intensity from 0.5 to 5.5 and hedonic tone from −3.5 to 1.

Proposed Structures	ANN Schematic
8-10-0-1 8-15-0-1 8-2-0-1 8-3-0-1 8-3-2-1 8-3-3-1 8-5-0-1 8-5-2-1 8-5-3-1 8-5-4-1 8-5-5-1	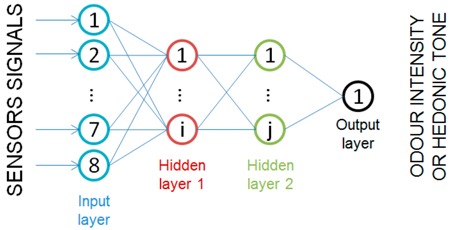

**Table 4:** Determined RMSECV and R^2^ parameters for artificial neural network characterized by different structure with tanh activation function for 150 samples of two-, three-, four- and five-component aqueous mixtures exhibiting odour intensity from 0.5 to 5.5 and hedonic tone from −3.5 to 1.

Odour Intensity			Hedonic Tone		
**ANN Structure**	**RMSECV**	**R^2^**	**ANN Structure**	**RMSECV**	**R^2^**
8-10-0-1	0.55	0.432	8-10-0-1	0.73	0.822
8-15-0-1	0.54	0.437	8-15-0-1	2.86	0.312
8-2-0-1	0.11	0.880	8-2-0-1	0.51	0.875
8-3-0-1	0.10	0.896	8-3-0-1	0.58	0.858
8-3-2-1	0.11	0.886	8-3-2-1	0.59	0.857
8-3-3-1	0.10	0.899	8-3-3-1	0.60	0.852
8-5-0-1	0.12	0.877	8-5-0-1	0.67	0.834
8-5-2-1	0.15	0.837	8-5-2-1	0.38	0.908
8-5-3-1	0.20	0.783	8-5-3-1	0.51	0.875
8-5-4-1	0.20	0.787	8-5-4-1	0.68	0.833
8-5-5-1	0.27	0.710	8-5-5-1	0.78	0.809

**Table 5:** Determined RMSECV and R^2^ parameters for artificial neural network characterized by different structure with activation function Leaky ReLUs with parameter a=0.03 for 150 samples of two-, three-, four- and five-component aqueous mixtures exhibiting odour intensity from 0.5 to 5.5 and hedonic tone from −3.5 to 1.

Odour Intensity			Hedonic Tone		
**ANN Structure**	**RMSECV**	**R^2^**	**ANN Structure**	**RMSECV**	**R^2^**
8-10-0-1	0.28	0.708	8-10-0-1	0.78	0.824
8-15-0-1	0.16	0.834	8-15-0-1	0.86	0.792
8-2-0-1	0.13	0.866	8-2-0-1	0.49	0.895
8-3-0-1	0.11	0.880	8-3-0-1	0.42	0.895
8-3-2-1	0.10	0.890	8-3-2-1	0.28	0.932
8-3-3-1	0.11	0.884	8-3-3-1	0.32	0.921
8-5-0-1	0.11	0.883	8-5-0-1	0.28	0.930
8-5-2-1	0.13	0.866	8-5-2-1	0.30	0.927
8-5-3-1	0.10	0.888	8-5-3-1	0.33	0.919
8-5-4-1	0.16	0.828	8-5-4-1	0.36	0.910
8-5-5-1	0.11	0.882	8-5-5-1	0.32	0.921

**Table 6:** Root mean square error of prediction determined using selected artificial neural networks for odour intensity and hedonic tone of two-, three-, four- and five-component odorous mixtures.

Amount of Mixture Components	RMSEP	
	Odour Intensity (8-3-3-1 tanh)	Hedonic Tone (8-3-2-1 Leaky ReLUs)
2	0.13	0.28
3	0.16	0.33
4	0.19	0.36
5	0.20	0.47

**Table 7:** Comparison of information obtained with sensory analysis and electronic nose measurements concerning occurrence of odour interactions in two-, three-, four- and five-component mixtures.

Amount of Mixture Components	Odour Intensity Interactions		Hedonic Tone Interactions	
	Sensory Analysis—Theoretical	ANN—Theoretical	Sensory Analysis—Theoretical	ANN—Theoretical
2	6	6 (100%)	6	5 (83%)
3	8	7 (88%)	9	7 (78%)
4	9	8 (89%)	11	8 (73%)
5	12	9 (75%)	14	9 (64%)
